# Mechanical Stimulation (Pulsed Electromagnetic Fields “PEMF” and Extracorporeal Shock Wave Therapy “ESWT”) and Tendon Regeneration: A Possible Alternative

**DOI:** 10.3389/fnagi.2015.00211

**Published:** 2015-11-09

**Authors:** Federica Rosso, Davide E. Bonasia, Antonio Marmotti, Umberto Cottino, Roberto Rossi

**Affiliations:** Department of Orthopaedics and Traumatology, AO Mauriziano Umberto ITorino, Italy

**Keywords:** tendon, tendinopathy, mechanical stimulation, extracorporeal shockwaves therapy, pulsed electromagnetic fields, tendon regeneration

## Abstract

The pathogenesis of tendon degeneration and tendinopathy is still partially unclear. However, an active role of metalloproteinases (MMP), growth factors, such as vascular endothelial growth factor (VEGF) and a crucial role of inflammatory elements and cytokines was demonstrated. Mechanical stimulation may play a role in regulation of inflammation. *In vitro* studies demonstrated that both pulsed electromagnetic fields (PEMF) and extracorporeal shock wave therapy (ESWT) increased the expression of pro-inflammatory cytokine such as interleukin (IL-6 and IL-10). Moreover, ESWT increases the expression of growth factors, such as transforming growth factor β(TGF-β), (VEGF), and insulin-like growth factor 1 (IGF1), as well as the synthesis of collagen I fibers. These pre-clinical results, in association with several clinical studies, suggest a potential effectiveness of ESWT for tendinopathy treatment. Recently PEMF gained popularity as adjuvant for fracture healing and bone regeneration. Similarly to ESWT, the mechanical stimulation obtained using PEMFs may play a role for treatment of tendinopathy and for tendon regeneration, increasing *in vitro* TGF-β production, as well as scleraxis and collagen I gene expression. In this manuscript the rational of mechanical stimulations and the clinical studies on the efficacy of extracorporeal shock wave (ESW) and PEMF will be discussed. However, no clear evidence of a clinical value of ESW and PEMF has been found in literature with regards to the treatment of tendinopathy in human, so further clinical trials are needed to confirm the promising hypotheses concerning the effectiveness of ESWT and PEMF mechanical stimulation.

## Introduction

Tendon disorders include both acute and chronic diseases, such as tendinopathy. It is know that the tendon tissue is poorly cellularized, with 5% of the normal tissue occupied by tenocytes that produce the extracellular matrix (ECM), based on type I collagen. Furthermore, along with tenocytes, the human tendons are also composed by tendon stem/progenitor cells (TSPCs), that guarantee to the tendon the ability to repair and regenerate and help in maintaining the homeostasis (Bi et al., 2007).

There is a debate on the role of inflammation in the production of degenerative changes in the tendon tissue (Abate et al., 2009; Cook and Purdam, 2009). However, recent new findings demonstrated the presence of inflammatory elements in pathologic tendons, as well as the activation of matrix metallo-proteinases, and the involvement of mediators such as substance P, vascular endothelial growth factor (VEGF), and cyclo-oxigenase type II (COX2; De Mattei et al., 2003). Tendon healing normally occurs in three different phases: the acute inflammatory phase, for up to 3–7 days, in which the neo-angiogenesis occurs, followed by the proliferative phase, for up to 21 days, when intrinsic cell proliferation of epitenon and endotenon tenocytes and extrinsic invasion of cells from the surrounding sheath and synovial occur, simultaneously with collagen, fibronectin, and elastin production, and the third remodeling phase, for up to 2 years (De Palma and Rapali, 2006; Abate et al., 2009). Different studies evaluated the molecular mechanisms that promote the healing process, such as metalloproteinases (MMPs) and MMPs with thrombospondin motifs (ADAMTs), in association with their tissue inhibitors (TIMPs; Sharma and Maffulli, 2005). Particularly MMP-9 and MMP-13 participate in collagen degradation only, while MMP-2, MMP-3, and MMP-14 are involved in both collagen degradation and remodeling (Riley et al., 2002). Indeed, different growth factors may be involved in neo-vascularization and stimulation of fibroblast and tenocytes, such as VEGF, TGF-β1, fibroblast growth factors (FGFs), and Scleraxis (Scx; Sharma and Maffulli, 2006). Furthermore, Nitric Oxide (NO) may also be involved in the healing process (Murrell, 2007).

In this scenario, different conservative treatments were proposed to treat tendon degeneration and tendinopathy. In this manuscript, we will analyze the rationale of mechanical stimulation by Extracorporeal shock wave (ESW) and Pulsed electromagnetic fields (PEMF) for tendon regeneration, and its possible role in the treatment of different tendinopathies.

## Extracorporeal shock wave therapy (ESWT) for tendon's pathology

### Shock waves' definition and mechanism of action

A shock wave is a special, non-linear type of pressure wave with a short rise time (around 10 μs) and a frequency ranging from 16 to 20 MHz (Ogden et al., 2001). These waves have a positive and negative (low-pressure) phase. In the first phase, ESW may hit an interface, with their reflection, or may gradually pass and become absorbed. The second phase causes cavitation at the tissue interfaces, with bubbles formation that subsequently implode, generating a second wave (Ogden et al., 2001). The propagating wave increase the tissue density and, as a consequence, transmit direct mechanical perturbations to the tissue with effects on cell membrane polarization, radical formation, cell proliferation, and growth factor production. Low energy ESW with a shock number ranging from 200 to 300 impulses seem most suited for enhancing cell proliferation and metabolism and, subsequently, for clinical applications.

There are two type of shockwave therapy: the focused shockwave therapy (FSWT) and the radial shockwave therapy (RSWT). *Focused shockwaves* are characterized by a pressure field that converges at a selected depth in the body tissues, where the maximal pressure is reached. FSWT can be generated using three methods: electro hydraulic (EH), electromagnetic (EM), and piezoelectric (PE). In all the cases the wave is generated in water, because of the acoustic impedance of water and biologic tissue are similar (van der Worp et al., 2013). The difference between the three methods of generation is the time at which the shockwave forms (Coleman and Saunders, 1989). RSWs are characterized by a diverging pressure field, which reach the maximal pressure at the source, and they are not generated in water (van der Worp et al., 2013). A recent *in vitro* study of Notarnicola et al. pointed out the negative effect of RWS on bone metabolism (Notarnicola et al., 2012), while some evidences in literature suggests a positive effect of RWS on the enthesis of plantar fascia and on the degenerated areas of Achilles tendon structure (van der Worp et al., 2013).

The supposed mechanism of action of ESW rely on conformational changes in membrane proteins, such as the integrins and, subsequently, on intracellular signal generation that modify gene expression and release of growth factors. Indeed, shock waves-induced repair phenomenon is observed in presence of increased level of IL-6, which is able to stimulate fibroblast production of collagen and ECM components (Waugh et al., 2015). A transient increase of IL-1b expression and a prolonged increase of IL-8 one was also described, consistent with a modulation of initial inflammatory phase. Following these observations, it is not surprising that ESW may stimulate matrix metalloproteinase (MMP) activity. Specifically, an increased expression of MMP-2 and the pro-MMP-9 was demonstrated. Considering the well-known low level of basal repair and MMP activity (i.e., MMP-9) in degenerative tendinopathy, these concepts support the statement that ESWT may also favor tendon repair by increasing pro-MMP forms availability, allowing for greater pathological tendon remodeling by ECM-degrading enzymes and, ultimately, favoring tendon tissue regeneration. Indeed, MMPs are also capable to activate latent TGF-β sequestered in the ECM. Nevertheless, a great inter-individual variability in the response of MMP to ESW was observed. This may represent a possible biological explanation for the variations in clinical success rates of ESWT in different tendinopathies and may be a clue for recognize and identify the population of responders and non-responders to ESWT.

Vetrano et al. demonstrated that ESWT up-regulates the expression of collagen (mainly type I) and stimulate cell proliferation in primary cultured human healthy tenocytes (Vetrano et al., 2011). However, the same group compared the results of ESWT (at the dose of 0.14 mJ/mm^2^) in cultured healthy and pathologic tenocytes, demonstrating that Scx and collagen type I were significantly diminished in the pathologic tenocytes cultures. These results indicate that the natural trigger for healing may be delayed by ESW treatment, in order to promote cellular repair (Leone et al., 2012). Pre-clinical studies showed that ESW are able to increase VEGF, VEGF receptor Flt-1, endothelial nitric oxide synthase (eNOS), and proliferating cell nuclear antigen (PCNA) expression, consistent with the initial neo-vascularization process. Improved blood supply and early vascularity is associated with the initial leukocyte infiltration and the subsequent metabolism of the fibers in the putative tendon pathological area by means of ECM-degrading enzymes (Bosch et al., 2009). This early phase is followed by an ESW-driven transitory increase in TGF-β1 expression, later followed by persistent IGF-I expression, that leads to a controlled inhibition of macrophages-induced ECM degradation and inflammation and an enhanced ECM and collagen type I synthesis (Visco et al., 2014). Tendon cells proliferation was also associated in this repair sequence, as well as endogenous lubricin production by fibroblasts and tenocytes following growth factors stimulation (i.e., TGF-β1; Zhang, 2011). The ultimate result of ESWT is a simulation of cell metabolism, which may induce healing process in injured areas of the tendons (Chen et al., 2004).

Recently, soft-focused ESWT was proposed, in order to deliver the energy in a larger area (Kuo et al., 2009). In the *in vitro* study of, de Girolamo et al. the effect of soft-focused ESWT were evaluated on a primary culture of healthy human tendon cells in adherent monolayer culture. The rationale of the study was to maintain cell-to-cell contacts and cell interactions with the ECM during ESWT, as it is a crucial point in the mechano-trasduction process. Furthermore, physical forces, such as soft-focused ESWT, may influence conformational changes in membrane proteins, such as integrines, resulting in an intra-cellular signal with a modification of gene expression and growth factors release (de Girolamo et al., 2014). At the molecular level, the authors evaluated the relationship between IL-1β and the production of MMPs, considered to be responsible for ECM degradation and tendon degeneration (Clegg et al., 2007). Both MMP-3 and 13 were not influenced by soft-focused ESW exposure, suggesting that the increased levels of IL-1β were not correlated with the ECM degradation. On the other hand, an increased expression of SCX, collagen type I genes (regulated by SCX expression), TGF-β, anti-inflammatory cytokines IL-6 and Il-10 and VEGF (stimulated by IL-6 and IL-10) was observed during the first 7 days after exposure to soft-focused ESW, although SCX transcription decreased rapidly during the first 4 days. All these results suggest that soft-focused ESWT may positively modulate the initial beneficial inflammatory phase of the tendon healing process (Visco et al., 2014) and “normalize” the anabolic activities of tendon cells.

Although the lack of sure evidence, ESWT may also be efficient in reducing calcifications in tendon structure (van der Worp et al., 2013).

### Clinical results of ESWT in tendon's pathology treatment

ESWT is reported to be an effective treatment in different chronic tendon pathologies.

### Shoulder

In 2014 Bannuru et al. published a systematic review on 28 Randomized Controlled Trials (RCT) comparing high-energy vs. low-energy ESWT or placebo for treatment of calcific or non-calcific tendinitis of the shoulder. The authors concluded that high-energy ESWT was significantly better than placebo in reducing pain and improving function in calcific tendinopathy, while no differences were detected between ESWT and placebo in non-calcific tendinopathy (Bannuru et al., 2014).

Huisstede et al. in 2011, in a systematic review, included 17 RCTs about ESWT vs. placebo in calcific and non-calcific rotator cuff tendinopathy. The authors concluded for strong evidence toward the best effectiveness of high-ESWT in calcific rotator cuff tendinopathy. Furthermore, no differences between the treatments were found in non-calcific rotator cuff tendinopathy (Huisstede et al., 2011). Similar results were reported in the systematic review by Harniman et al. (2004). The single studies are summarized in Table 1.

**Table 1:** **Summary of literature studies on ESWT, Extra-corporeal Shock Waves Therapy; RSWT, radial shockwave therapy**.

**Authors and Year**	**Pathology**	**Number of patients**	**Level**	**Type ESWT**	**Follow-up**	**Outcomes**
**SHOULDER**						
Loew et al., 1999	Calcific tendinitis of the shoulder	195 (80 divided in 4 groups with different regimens, 115 divided into one or two session)	II	- High-ESWT EFD: 0.30 mJ/mm^2^ (high) one session—double - Low-ESWT: 0.1 mJ/mm^2^(high) one session—double Control (no treatment; *n* = 20)	Not reported	The results showed energy-dependent success. With 58% of pain relief after two high-energy session
Schmitt et al., 2001	Non-calcific supraspinatus tendinitis	40	II	- High-ESWT 0.11 mJ/mm^2^(*n* = 20) - Sham ESWT (*n* = 20)	12 weeks	Increased function and a reduction of pain in both groups (*p* < or = 0.001). The authors did not recommend ESWT for the treatment of tendinitis of supraspinatus
Speed et al., 2002a	Non-calcific supraspinatus tendinosis6	74	II	- ESWT: 0.12 mJ/mm^2^(medium; *n* = 34) - ESWT: minimum: 0.04 mJ/mm^2^ (low; *n* = 40)	Not reported	No significant difference between the treatments in terms of pain. The authors concluded on no benefit of ESWT in patients with non-calcific tendonitis
Haake et al., 2002	Calcific tendinitis of the supraspinatus	50	II	- ESWT: focus on calcific deposit: 0.78 mJ/mm^2^(high; *n* = 25) - ESWT: focused on tuberculum majus: 0.78 mJ/mm^2^ (high; *n* = 25)	1 year	Significantly better Constant and Murley score in ESWT at the calcified area under fluoroscopic control
Pan et al., 2003	Calcific tendinitis of the shoulder	63	II	- High-ES WT 2 Hz 2000 shock waves, 2 sessions, 14 days apart 0.26–0.32 mJ/mm^2^(*n* = 33) - TENS 3x/week 20 min for 4 weeks (*n* = 30)	6 months	Better outcomes VAS and Constant score in the ESWT group compared to TENS group
Gerdesmeyer et al., 2003	Calcific rotator cuff tendinopathy	96	II	- High-ESWT (1500 pulses 0.32 mJ/mm^2^; *n* = 48) - Sham ESWT (*n* = 48)	1 year	High-ESWT and low-ESWT provided a beneficial effect on pain, function and calcifications' size. However, high-ESWT appeared to be superior compared to low-ESWT
Perlick et al., 2003	Calcific tendinitis of the shoulder	80	II	- ESWT: 0.23 mJ/mm^2^(medium; *n* = 40) - ESWT: 0.42 mJ/mm^2^ (high; *n* = 40)	1 year	Improvement in Constant and Murley scores. However, the disintegration of calcific deposits is dose-dependent
Peters et al., 2004	Calcific tendinosis of the shoulder	61	II	- High level ESWT: 0.44 mJ/mm^2^ (*n* = 31) - Medium level ESWT: 0.15 mJ/mm^2^ (*n* = 30)	6 months	ESWT in calcific tendinitis of the shoulder is very effective, without significant side effects at 0.44 mJ/mm^2^
Cosentino et al., 2004	Chronic calcific tendinitis of the shoulder	135	IV	ESWT 0.03 mJ/mm^2^ (4 sessions)	1 month	Improvement in the Constant and Murley score, with partial resorption of the deposits in 44.5% of patients, and complete resorption in 22.3% of patients
Krasny et al., 2005	Calcific supraspinatus tendinitis	80	II	- High-ESWT plus Ultrasound-guided needling (*n* = 40) - High-ESWT only (200 impulses followed by 2500 pulses, 0.36 mJ/mm^2^(*n* = 40)	4.1 months (average)	Ultrasound-guided needling in combination with high-ESWT is more effective compared to ESWT alone, with higher rates of deposits elimination, better clinical results and lower need for surgery
Sabeti-Aschraf et al., 2005	Calcific tendinitis of the shoulder	50	II	- ESWT: 0.08 mJ/mm^2^ Point of max tenderness (*n* = 25) - ESWT: 0.08 mJ/mm^2^Point of max tenderness by computer-assisted navigation device (*n* = 25)	12 weeks	Both groups had significant improvements in the Constant and Murley score and VAS score. However, the navigation group showed better results
Moretti et al., 2005	Rotator cuff calcifying tendinitis	44	IV	Four sessions of medium-ESWT (0.11 mJ/mm^2^) ESWT administered with an electromagnetic lithotripter	6 months	70% of satisfactory functional results. Disappearance of the deposits in 50% of the cases
Cacchio et al., 2006	Calcific tendinitis of the shoulder	50	II	- ESWT 4 sessions at 1-week intervals, with 25,00 pulses per session, 0.10 mJ/mm^2^ (*n* = 25) - ESWT 4 sessions at 1-week intervals, total number of pulses: 25 (*n* = 25)	6 months	Better functional results in the RSWT group
Albert et al., 2007	Calcific tendinitis of the shoulder	80	II	- ESWT: max 0.45 mJ/mm^2^(high; *n* = 40) - ESWT: 0.02-0.06 mJ/mm^2^ (low; *n* = 40)	110 days	High-ESWT group had significant better results, but with the calcific deposit unchanged in size in the majority of patients
Hsu et al., 2008	Calcific tendinitis of the shoulder	46	II	- High-ESWT: 0.55 mJ/mm^2^(*n* = 33 - Sham ESWT (*n* = 13)	1 year	No significant difference between Gärtner type I and type II groups in the Constant score (*P* >.05). ESWT are effective in the treatment of calcific tendinitis with negligible complication
Rebuzzi et al., 2008	Calcific tendinitis of the supraspinatus	46	IV	- Arthroscopic extirpation (*n* = 22) - Low - ESWT (*n* = 24)	24 months	No differences in UCLA scores. ESWT have similar results compared to arthroscopy
Schofer et al., 2009	Non-calcific shoulder tendinopathy	40	II	- High-ESWT-1 0.78 mJ/mm^2^(*n* = 20) - High-ESWT-2 0.33 mJ/mm^2^(*n* = 20)	12 weeks	Statistically significant improvement in both groups, without statistically significant differences between high-ESWT and low-ESWT
Ioppolo et al., 2012	Supraspinatus Calcifying Tendinitis	46	II	- ESWT at an energy level of 0.20 mJ/mm^2^ - ESWT at an energy level of 0.10 mJ/mm^2^	6 months	Better results in the first group of treatment (Constant Murley Scale = CMS)
Galasso et al., 2012	Non-calcifying supraspinatus tendinopathy	20	II	- ESWT - sham control group	12 weeks	ESWT groups showed better CMS score, without any side effect
**PATELLAR TENDINOPATHY**						
Peers et al., 2003	Chronic patellar tendinopathy	27	III	- Surgical treatment (*n* = 13) - ESWT (*n* = 14)	6 months	ESWT showed comparable outcomes compared to surgery
Taunton and Khan, 2003	Chronic patellar tendinopathy	30	II	- ESWT (*n* = 20) - ESWT with energy-absorbing pad (*n* = 10)	Not reported	ESWT is effective in adjunction with eccentric exercises in treating patellar tendinopathy
Wang et al., 2007	Chronic patellar tendinopathy	50	II	- ESWT (0.18 mJ/mm^2^ energy flux density; *n* = 27) - Conservative treatment (*n* = 23)	2-3 years	ESWT is more effective compared to conservative treatment
Vulpiani et al., 2007	Jumper's knee	73	IV	- ESWT (4 sessions 1500–2500 impulses,energy varying between 0.08 and 0.44 mJ/mm^2^)	Not reported	Satisfactory outcomes in ESWT treatment for jumper's knee
Zwerver et al., 2010	Severe patellar tendinopathy	19	IV	Patient guided Piezo-electric, focused ESWT	3 months	Patient guided Piezo-electric ESWT without local anesthesia is a safe and well-tolerated treatment for severe patellar tendinopathy
Zwerver et al., 2011	Patellar tendinopathy in athletes	62	I	- ESWT (*n* = 31) - Sham ESWT (*n* = 31)	1 year	No benefit of ESWT over placebo in treatment of patellar tendinopathy in in-season athletes
Furia et al., 2013	Chronic patellar tendinopathy	66	III	- Radial low-ESWT (*n* = 33) - Conservative treatment (*n* = 33)	1 year	The percentage of “excellent” functional outcomes was significantly higher in the ESWT group
**ELBOW PATHOLOGY**						
Rompe et al., 2001	Chronic lateral epicondylitis of the elbow	30	II	- ESWT (0.16 mJ/mm^2^) - ESWT (0.16 mJ/mm^2^) plus cervical manual therapy	1 year	Each group showed significant improvement in the pain and functional scores. The authors concluded that ESWT may be an effective conservative treatment method for unilateral chronic tennis elbow
Maier et al., 2001	Chronic lateral tennis elbow	42	IV	ESWT	18.6 months	Good clinical performances after ESWT. Male patients performed better than female ones. In female patients, Magnetic Resonance Imaging (MRI) may predict the results of ESWT
Speed et al., 2002b	Lateral epicondylitis	75	II	- ESWT at 0.12 mJ/ mm^2^ - Sham therapy	1 year	No significant difference between the groups, concluding that the placebo effect of ESWT may be considerable
Melegati et al., 2004	Lateral epicondylitis	41	II	- ESWT (Lateral tangential focusing) - ESWT (tangential focusing)	Not reported	No differences between the techniques
Furia, 2005	Chronic lateral epicondylitis	36	IV	ESWT	Not reported	77.8% were rated excellent or good on the Roles and Maudsley scale
Chung et al., 2005	Chronic lateral epicondylitis	60	II	- ESWT + stretching program - Sham therapy	1 year	No differences in clinical outcomes
Staples et al., 2008	Lateral epicondylitis	68	II	−3 ESWT treatments - 3 treatments at a subtherapeutic dose	6 months	Little evidence in favor of ESWT in the treatment of lateral epicondylitis
Radwan et al., 2008	Resistant tennis elbow	46	II	- high - ESWT (0.22 mJ/mm^2^; *n* = 29) - Percutanous tenotomy (*n* = 27)	1 year	Excellent and good results were achieved in 65.5% of patients in ESWT group and 74.1% in the percutanous group
Gunduz et al., 2012	Lateral epicondylitis	59	II	- Physical therapy - A single corticosteroid injection - ESWT	6 months	All the treatment had favorable effects on pain and grip strength in the early period
Lee et al., 2012	Medial and lateral epicondylitis	22	III	- ESWT group (0.06–0.12 mJ/mm^2^; *n* = 12) - local steroid injection group (*n* = 10)	8 weeks	Both the treatments were effective for medial and lateral epicondylitis
Notarnicola et al., 2014	Epicondylitis	26	IV	ESWT	Not reported	Progressive improvement in pain during the follow-up, with decrease in grip strength, especially in the dominant limb
Trentini et al., 2015	Lateral epicondylitis	36	IV	Focused ESWT	24.8 months	75.7% of positive response. Focal ESWT is a valuable and safe solution in case of lateral epicondylitis, both in newly diagnosed and previously treated cases
**FOOT PATHOLOGY**						
Furia, 2006	Insertional Achilles tendinopathy	88	II	- ESWT group; 0.21 mJ/mm^2^; total energy flux density, 604 mJ/mm^2^ (*n* = 35) - Non-operative therapy (*n* = 33)	1 year	Better Roles and Maudsley results in the ESWT group. NO differences if a local anesthesia was performed or not before the ESWT session
Rasmussen et al., 2008	Chronic Achilles tendinopathy	48	II	- active ESWT - sham ESWT	12 weeks	Better results in the active ESWT group
Vulpiani et al., 2009	Achilles tendinopathy	115	IV	ESWT (0.08 and 0.40 mJ/mm^2^)	1 year	76% of satisfactory results at the last follow-up
Saxena et al., 2011	Achilles tendinopathy	74	IV	ESWT	1 year	74.8% of patients improved 1 year after surgery, with significant improvement of the Roles and Maudsley score
Kim et al., 2015	Plantar fasciitis	10	IV	ESWT	6 months	Decreased plantar fascia thickness, spasticity, and pain and increased gait ability after ESWT

### Patellar tendinopathy

Van Leeuwen et al. in 2009 published a systematic review describing the results of ESWT in patellar tendinopathy, collecting seven RCTs on ESWT vs. placebo. The authors concluded on positive results using ESWT in treating patellar tendinopathy, but most of the studies had different frequency of treatments, application, and shockwave generation, energy level and method of localization (van Leeuwen et al., 2009). However, Zwerver et al. in 2011 published a RCT on 62 symptomatic athletes affected by patellar tendinopathy treated either with ESWT or placebo, concluding for no beneficial effects of ESWT (Zwerver et al., 2011). Due to these conflicting reports, further studies are needed to clarify the value of ESWT for patellar tendinopathy. Again, the most recent studies are summarized in Table 1.

### Elbow

Buchbinder et al. in 2005 published a systematic review regarding the effectiveness and safety of ESWT for lateral elbow pain. The authors included nine trials, randomizing 1006 participants to ESWT or placebo and one trial including 93 participants randomized into ESWT or steroid injection. They concluded that steroid injections were more effective compared to ESWT (Buchbinder et al., 2005). Recently, Trentini et al. reported the results on 36 patients affected by lateral epicondylitis and treated with focal ESWT. At a mean follow-up of 24.8 months, the authors described a positive response to the treatment in 75.7% of the patients (Trentini et al., 2015). These studies outlines that, the clinical efficacy of ESWT for the treatment of medial and lateral epycondylitis is still controversial, as shown in the papers reported in Table 1.

### Foot pathology

In 2013 Al-Abbad et al. published a systematic review including six studies (4 RCTs) and evaluating the efficacy of ESWT for Achilles tendinitis treatment. The authors concluded that ESWT was effective for Achilles tendinopathy at a minimum 3 months' follow-up (Al-Abbad and Simon, 2013). Other studies pointed out similar conclusions as shown in Table 1.

Recently, Kim et al. analyzed the efficacy of ESWT also for plantar fasciitis in stroke patients, reporting on reduced tension in the plantar fascia, and observing pain relief and improved gait ability (Kim et al., 2015; Table 1). Their results are similar to other recent studies (Park et al., 2014; Yin et al., 2014; Gollwitzer et al., 2015; Konjen et al., 2015; Mardani-Kivi et al., 2015), and allows for considering ESW a precious therapeutic modality for treating acute or recalcitrant plantar fasciitis with acceptable success rate ranging from 50% to 80%, at least at a short term follow-up.

## Pulsed electromagnetic fields (PEMF) for tendon's pathology

### Pulsed electromagnetic fields (PEMF) definition and mechanism of action

PEMF are characterized by frequencies at the low end of the electromagnetic spectrum, ranging between 6 and 500 Hz (Bassett, 1989). Another feature of PEMF waveforms is the rate of change: higher rate of changes (Tesla/seconds) are able to induce biological currents in the tissue, with peculiar biological effects (Juutilainen and Lang, 1997). Furthermore, it was demonstrated that low-frequency fields are non-ionizing and athermal (Rubik, 1997).

Different types of waveforms were associated to PEMF: asymmetric, biphasic, sinusoidal, quasi-rectangular, or quasi-triangular in shape (Bassett, 1989). In 1979, the Food and Drug Administration (FDA) approved both quasi-rectangular and quasi-triangular PEMF as safe and effective for the treatment of fractures and their sequelae (Bassett, 1989).

There are two methods in which PEMF can be applied to biological tissues: capacitive or inductive coupling. Capacitive coupling does not involve any contact with the body. However, in direct capacitive coupling, an electrode has to be placed on the skin of the opposite side (Trock, 2000). On contrary inductive coupling does not require the electrodes to be in direct contact with the skin, because the magnetic field produces an electric field that, in turn, produces a current in the conductive tissues of the body (Stiller et al., 1992; Trock, 2000).

Similarly to ESW, PEMF are physical stimuli that produce membrane disturbances and activation of multiple intracellular pathways. Indeed, formation of lipidic “nanopores” in the plasma membrane following PEMF exposure may explain the conduction of ions into the cell from the extracellular space, specifically Calcium ions (Ca). Furthermore, a direct effect of PEMF on phospholipids within the plasma membrane has been postulated, with a subsequent production of several second messengers, initiating multiple intracellular signal transduction pathways, as well as a further activation of protein kinase C (Semenov et al., 2013; Tolstykh et al., 2013).

Nevertheless, PEMFs have been recently connected to other cell activation pathways. In particular, the ligand-independent activation of epidermal growth factor receptor (EGFR) and other members of the receptor tyrosine kinase family were observed, with subsequent stimulation of intracellular signaling as the MAPK (mitogen-activated protein kinases)/ERK (extracellular signal-regulated kinases) pathway, with subsequently activation of intracellular mitogenic pathway (Wolf-Goldberg et al., 2013).

Recent studies outlined the expression of canonical Wnt signaling proteins (Wnt1/ lipoprotein receptor-related protein 5 LRP5/beta-catenin) in cell derived from a mesenchymal lineage and exposed to PEMF (Jing et al., 2013; Zhou et al., 2015). At this regard, the presence of beta-catenin seems particularly important with respect to other settings, in which this protein is linked to cell plasticity and proliferation, as in the superficial zone of articular cartilage (Yasuhara et al., 2011; Marmotti et al., 2013).

Finally, the exposure to PEMF induces early up-regulation of adenosine receptor A_2A_ and A_3_. Adenosine receptor A_2A_ and A_3_ are able to reduce PGE2 and pro-inflammatory cytokine IL-6 and IL-8 release and to inhibit the activation of transcription factor NF-kB, a key regulator of inflammatory responses (Vincenzi et al., 2013), as well as to positively interfere in several cell activities as cell proliferation (Varani et al., 2008).

Taken together, all these observations suggest a possible role of PEMF for “tenocyte activation” (Dingemanse et al., 2014; de Girolamo et al., 2015). This may be achieved by two main mechanisms: (i) limiting the catabolic effects of pro-inflammatory cytokines such as IL-1, IL-6, and IL-8 and (ii) increasing ECM production, cytokine release and cell proliferation (De Mattei et al., 2003; Fassina et al., 2006; Ongaro et al., 2012; de Girolamo et al., 2013). Recent studies of de Girolamo et al. (de Girolamo et al., 2013, 2015) demonstrated that human tendon cells proliferation was enhanced after PEMF treatment, and in particular, that a 1.5 mT-PEMF treatment was able to up-regulate SCX, VEGF-A, and COL1A1 gene expression. Moreover, the treated tendon cells showed, after 2 days, a higher release of IL-6, IL-10, and TGF-β. These effects are essential for tendon metabolism, inducing increased elastin and fibronectin production, increased cell proliferation and neo-angiogenesis.

### Clinical results of PEMF in tendon's pathology treatment

Despite the lack of recent literature sustaining a long-term positive effect of PEMF in treating shoulder and elbow tendon disorders (Uzunca et al., 2007; Bisset et al., 2011; Dingemanse et al., 2014), a positive effect of PEMF in reducing lateral epicondylitis pain was described at a short term follow-up (3 months). Moreover, several reports back in the 80's proposed a putative role in the treatment of rotator cuff disease and lateral epicondylitis (Binder et al., 1984; Devereaux et al., 1985). A very recent study by Osti et al. described a possible role of PEMF after rotator cuff repair as an adjuvant treatment, in order to reduce local inflammation, post-operative joint swelling and stiffness, and recovery time, as well as to induce pain relief. A significant short term (up to 5 months) positive effect was observed in PEMF treated patients, but the authors did not observe any clinical and functional improvement at a longer (2 years) follow-up (Osti et al., 2015).

Some suggestions for the use of PEMF for treating human Achilles tendon pathologies come only from preclinical animal studies. Strauch et al. in 2006 analyzed the effect of PEMF on the biomechanical strength of rat Achilles' tendons at 3 weeks after transection and surgical repair, with an increase in tensile strength of up to 69% 3 weeks after the surgery (Strauch et al., 2006). Previously, Lee et al. in 1997 analyzed the possible role of pulsed magnetic fields (PMF) and (PEMF) in the healing process of Achilles tendon inflammation in the rat (Lee et al., 1997).

## Conclusion

Mechanical stimulation by means of ESW and PEMF seems to be favorable for tendon regeneration in preclinical and *in vitro* studies. Indeed, a beneficial effect has been demonstrated on tendon resident cells at a cellular level. Increased cell proliferation and the production of the immuno-regulatory cytokines and growth and angiogenic factors was observed *in vitro*, consistent with an overall “tenocyte activation” favoring tendon healing process. Conversely, there is still a lack of strong evidences on ESW and PEMF when dealing with clinical settings. The efficacy of ESWT is demonstrated for the treatment of calcific rotator cuff tendinopathy, while it is still debated its role in the treatment of other tendon disorders, such as patellar tendinopathy and lateral epicondylitis. PEMF seem to exert positive clinical effects toward shoulder and elbow tendon disorders only at a short term follow up. While basic science research continues to show encouraging results, further *in vivo* human studies are undoubtedly necessary to confirm the clinical efficacy of mechanical stimulation by means of PEMF and ESW for the treatment of tendon disorders.

## Author contributions

FR Substantial contributions to the conception or design of the work, drafting the work, acquisition, analysis, or interpretation of data for the work. DB Substantial contributions to the conception or design of the work, revising the work. AM Substantial contributions to the conception or design of the work, revising the work. UC Substantial contributions to the conception or design of the work, revising the work. RR Revising the work, Final approval of the version to be published.

### Conflict of interest statement

The authors declare that the research was conducted in the absence of any commercial or financial relationships that could be construed as a potential conflict of interest.
